# RETRACTION: Correlation Between Acanthosis Nigricans Scoring Chart (ANSC) and Narrowband Reflectance Spectrophotometer in Assessing Severity of Acanthosis Nigricans

**DOI:** 10.1111/srt.70341

**Published:** 2026-02-26

**RETRACTION**: T. Treesirichod, C. Kritsanaviparkporn, P. Sangaphunchai, and S. Chansakulporn, “Correlation Between Acanthosis Nigricans Scoring Chart (ANSC) and Narrowband Reflectance Spectrophotometer in Assessing Severity of Acanthosis Nigricans,” *Skin Research and Technology* 29, no. 8 (2023): e13428, https://doi.org/10.1111/srt.13428.

The above article, published online on 30 July 2023 in Wiley Online Library (wileyonlinelibrary.com), has been retracted by agreement between *Skin Research and Technology*; and John Wiley & Sons Ltd. The article was accepted for publication following an insufficient peer review process that does not meet the standards of the journal's peer review policy. Furthermore, methodological concerns have been raised by Bitterman *et al*. (2023) that, in the editors' view, compromise the reliability of the results presented and conclusions drawn. The authors maintain that the methodological limitations described by Bitterman et al. were inherent to the assessment tool employed and should be interpreted within that context, disagreeing with the determination that these issues undermine the validity of the study's findings. This retraction is therefore issued as administrative due diligence to correct the scholarly record and alert readers to these deficiencies in the publication process.
